# Study of the Potential of the Capsule Shell Based on Natural Polysaccharides in Targeted Delivery of the L-Phenylalanine Ammonia-Lyase Enzyme Preparation

**DOI:** 10.3390/ph13040063

**Published:** 2020-04-09

**Authors:** Olga Babich, Lyubov Dyshlyuk, Alexander Prosekov, Svetlana Noskova, Oksana Ivina, Valery Pavsky, Svetlana Ivanova, Olga Bulgakova

**Affiliations:** 1 Institute of Living Systems, Immanuel Kant Baltic Federal University, A. Nevskogo Street 14, Kaliningrad 236016, Russia; olich.43@rambler.ru (O.B.); svykrum@mail.ru (S.N.); 2 Research Institute of Biotechnology, Kemerovo State University, Krasnaya Street 6, Kemerovo 650043, Russia; soldatovals1984@mail.ru (L.D.); pavva46@mail.ru (V.P.); 3 Natural Nutraceutical Biotesting Laboratory, Kemerovo State University, Krasnaya Street 6, Kemerovo 650043, Russia; 4 Laboratory of Biocatalysis, Kemerovo State University, Krasnaya Street 6, Kemerovo 650043, Russia; a.prosekov@inbox.ru; 5 Department of Information and Automated Production Systems, T.F. Gorbachev Kuzbass State Technical University, Vesenyaya Street, 28, Kemerovo 650000, Russia; k8enya@e-mail.com; 6 Department of General Mathematics and Informatics, Kemerovo State University, Krasnaya Street, 6, Kemerovo 650043, Russia; vmkemsu@mail.ru

**Keywords:** capsules, natural polysaccharides, hydrocolloids, enzyme preparation, L-phenylalanine ammonia-lyase, phenylketonuria

## Abstract

The treatment of classical phenylketonuria is currently represented by many new methods of disease management. A promising method is the use of the enzyme L-phenylalanine ammonia-lyase (PAL) in various forms. The widespread use of enzyme preparations in therapy is limited by a lack of understanding of the mechanisms and systems of the targeted transport of PAL into certain organs and tissues as a result of the incorporation of a drug into the carrier. To ensure the stability of enzymes during the delivery process, encapsulation is preferable, which, as a rule, ensures the preservation of the qualitative characteristics of the enzymes orally applied to the environmental effects of the gastrointestinal tract (acidity, temperature, oxidation, etc.). Capsule preparations showed sufficient stability in the model gastric fluids and sustained release of the drug in the simulated intestinal fluid. Currently, there is a wide range of polymers used for encapsulation. The use of natural sources in the production technology of capsule systems improves bioavailability, controls the release, and prolongs the half-life of active substances. The advantage of this method is that the used enzyme is completely protected by the cell membranes of the capsules, which preserve its stability in the aggressive environment of the gastrointestinal tract. Capsules were obtained on the basis of compositions of hydrocolloids of plant origin. The potential of the developed capsules for targeted delivery of the enzyme preparation was studied. The degradation of the encapsulated form of the PAL enzyme preparation was studied in vitro in model bio-relevant media simulating the gastric and intestinal environment. The dynamics of the breakdown of the capsule shell allow us to expect that the release of L-phenylalanine ammonia-lyase from capsules based on plant hydrocolloids will occur no earlier than reaching the upper intestines, where the interaction with the protein components of the consumed food products to neutralize phenylalanine should occur.

## 1. Introduction

Phenylketonuria (PKU) is an autosomal recessive genetic disease that occurs in approximately 1/16,000 births. Classical phenylketonuria is caused by gene mutations on the 12th chromosome, which encodes phenylalanine hydroxylase (PAH). L-phenylalanine ammonia-lyase (PAL) is required for the conversion of phenylalanine to tyrosine [1]. A decrease in the activity of phenylalanine hydroxylase leads to neurotoxic damage to the nervous system and irreversible brain changes. Phenylalanine-restricted nutrition is the only effective treatment. However, the considerable cost of the diet and strict control of the composition of the food does not allow patients to adhere to it for long periods [2,3]. In addition, treatment with special medical products is associated with the risks of nutritional deficiencies, especially for vitamin B12, vitamin D, calcium, iron, and unsaturated long-chain fatty acids [4,5,6]. This can lead to neurological problems and problems associated with bone density [7,8].

The search for additional therapeutic options in the treatment of patients with phenylketonuria is ongoing. The most promising is the use in the treatment of the enzyme ammonia-lyase [9,10,11,12] in the form of both injections and capsules/tablets, which breaks down phenylalanine to safe products. The most significant results were obtained by BioMarin Pharmaceutical Inc [13,14,15]. Despite many years of research into this disease, it was BioMarin Pharmaceutical Inc (https://www.biomarin.com) that commissioned a survey among PKU sufferers about their quality of life and expected hopes for developing a therapy. The survey showed that the majority of respondents are interested in the development of new drugs and treatment methods, preferring the oral administration of pharmaceutical preparations with the possibility of not following a strict diet. This confirms that the problem has not yet been resolved and remains relevant [16,17,18]. There are studies that present methods for producing PAL in the cultivation of the yeast *Rhodotorula* and other microbial cells [19,20,21], purification, drying of PAL [22,23], stabilization [24,25], the use of PAL [22,26], and so forth [27,28,29].

Many enzymes are sensitive to pH <5, therefore, preparations intended for oral administration should be covered with an enteric protective layer. This makes it possible to preserve the acid-sensitive preparations from irreversible breakdown and release active components only after passing through the stomach in the upper region of the small intestine, where safe pH values usually prevail—approximately between pH 5.5 and pH 8 [30]. In addition, enteric polymer coatings are acidic in nature. To compensate for the acidic environment of the enteric polymer membrane, it is necessary to use additional protection of its composition. In some cases, to ensure the stability of the buffer composition, the enzyme is represented in the form of microspheres or microcapsules coated with an enteric membrane [31]. Studies related to the optimization of the coating composition of enzymatic preparations are carried out continuously [32,33,34].

The development of a carrier that can stabilize and protect the enzyme (PAL) from inactivation in an aggressive gastrointestinal environment is important for the development of an effective oral dosage form. When stabilizing enzymes, encapsulation is preferred due to the fact that this method provides high enzyme stability under the influence of temperature and acidity of the medium, as well as exposure to destructive microflora and oxygen. An important advantage of capsule preparations should include the ability to ensure even dosing of the enzyme. Since the essence of encapsulation is to create a kind of shell around the enzyme, an important step is the selection of suitable material for it. The effectiveness and protection of the drug, and especially the enzyme, depend on the characteristics and stability of the capsule shells against the negative environmental factors of the gastrointestinal tract. Currently, there is a wide range of polymers used for encapsulation. Polymers must meet the following criteria: the material must be physically and materially accessible; the material must be nontoxic, without chemicals and additives that can affect the enzyme and the human body; the material must decompose under the action of the microbiota of the gastrointestinal tract, providing a slow release of the drug (PAL); the material must be able to rehydrate properly to ensure enzyme stability [35,36,37]. The most common capsule base materials are materials such as sodium alginate, pectin, chitosan, carrageenan, gelatin, agarose, xanthan gelatin mixture, various cellulose derivatives, and so forth [38,39,40,41,42].

PAL’s widespread application for the treatment of phenylketonuria is hindered by its instability in the aggressive environment of the gastrointestinal tract [27,43,44] and lack of systems for preserving its effectiveness and targeted transporting for direct interaction with phenylalanine. The strategy for protecting enzymes from acidic and proteolytic destruction is to combine them with polymers that protect the enzyme. The use of native biodegradable capsule/microcapsule systems can not only effectively prevent the degradation of basic materials in the body or biological environment, but also improve bioavailability, control the release, and extend the half-life of active substances [45]. Therefore, different types of natural polymers can be evaluated in terms of encapsulating the enzyme preparation and providing PAL protection. The aim of the work was to study the effect of natural polysaccharide capsules on the stability of the enzyme preparation L-phenylalanine ammonium-lyase.

## 2. Results

The results of studying the qualitative and microbiological characteristics of capsule shell samples based on natural polysaccharides (Table 1) are presented in Table 2 and Table 3. Significant differences in the characteristics of the capsules, except for the mass fraction of moisture, disintegration, and flowability, were not observed (*p* > 0.05). Mass, as a property of absorption and moisture retention, is obviously determined by the density of the substance used for the manufacture of capsules. According to the observation results [46], capsules made of natural polysaccharides retained their quality characteristics at a temperature from 4 °C to 25 °C for 6 months (*p* = 0.913), and at a temperature from 15 °C to 25 °C for 36 months (*p* = 0.894), for almost all capsule formulations. According to [47], the total number of aerobic microorganisms in drugs should not exceed 10^3^ CFU/g, the total number of yeast and mold fungi should be 10^2^ CFU/g, and bacteria of the *E. coli* group should be absent. Vegetable hydrocolloid capsule samples meet the requirements of microbiological purity standards.

**Figure 1:**
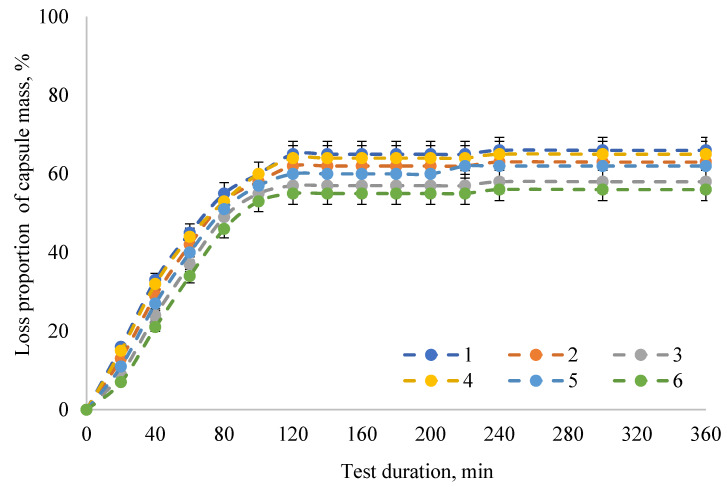
Dynamics of the in vitro degradation of the encapsulated form of the PAL enzyme preparation in distilled water: 1—samples of capsules made according to the formulation No. 1; 2—samples of capsules made according to the formulation No. 2; 3—samples of capsules made according to the formulation No. 3; 4—samples of capsules made according to the formulation No. 4; 5—samples of capsules made according to the formulation No. 5; 6—samples of capsules made according to the formulation No. 6. The data are expressed as mean ± SE (*n* = 3).

**Figure 2:**
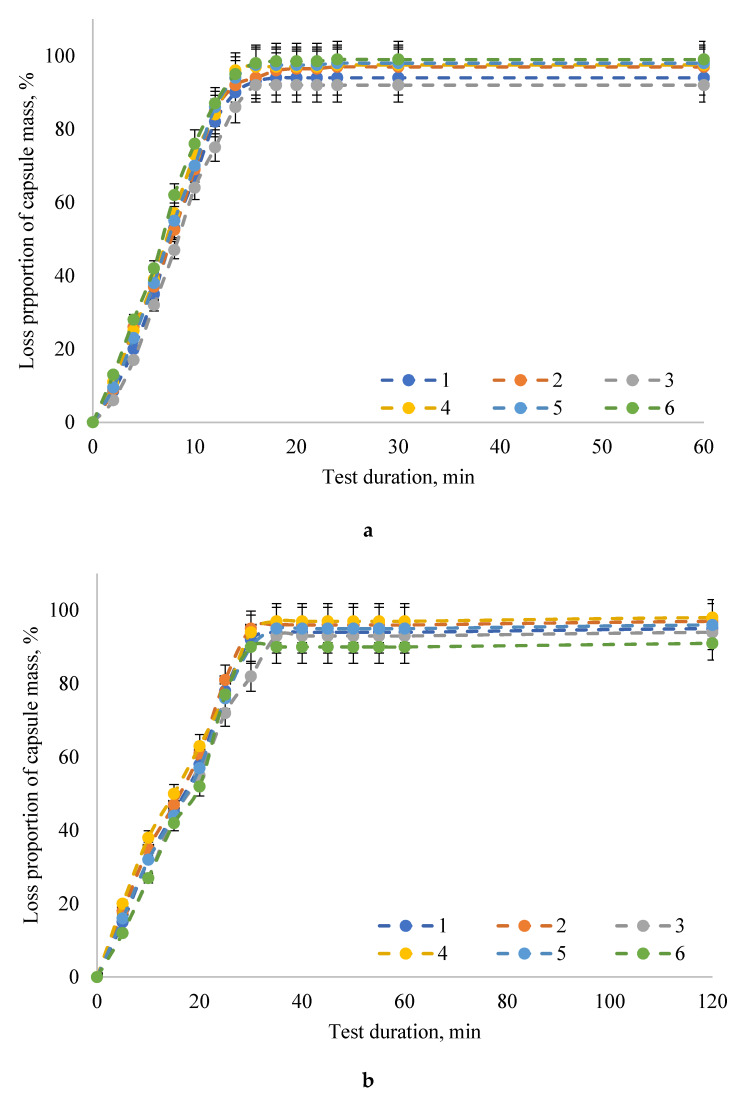
Dynamics of the in vitro degradation of the encapsulated form of the PAL enzyme preparation in intestinal model media (**a**) SIF and (**b**) FaSSIF: 1—samples of capsules made according to the formulation No. 1; 2—samples of capsules made according to the formulation No. 2; 3—samples of capsules made according to the formulation No. 3; 4—samples of capsules made according to the formulation No. 4; 5—samples of capsules made according to the formulation No. 5; 6—samples of capsules made according to the formulation No. 6. The data are expressed as mean ± SE (*n* = 3).

**Figure 3:**
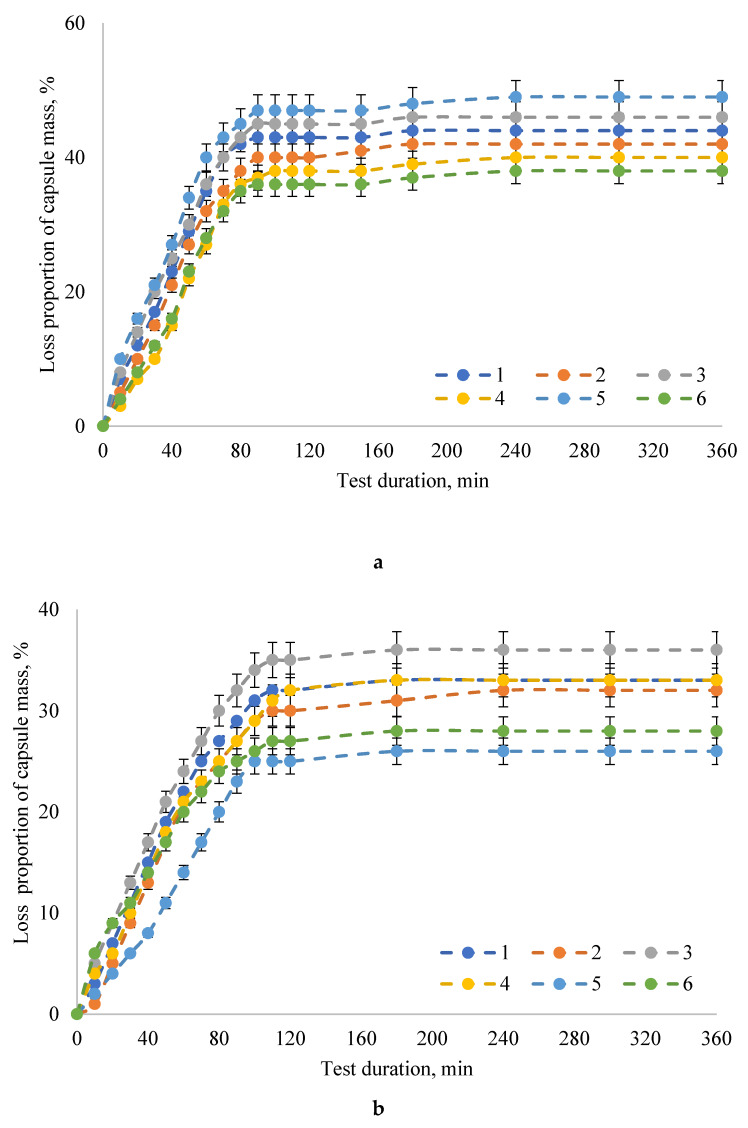
Dynamics of the in vitro degradation of the encapsulated form of the PAL enzyme preparation in gastric model media (**a**) SGF and (**b**) FaSSGF: 1—samples of capsules made according to the formulation No. 1; 2—samples of capsules made according to the formulation No. 2; 3—samples of capsules made according to the formulation No. 3; 4—samples of capsules made according to the formulation No. 4; 5—samples of capsules made according to the formulation No. 5; 6—samples of capsules made according to the formulation No. 6. The data are expressed as mean ± SE (*n* = 3).

The results obtained allow use of capsule samples from natural polysaccharides to create a capsule form of the enzyme preparation. The principal condition in this case is the possibility of delivering the encapsulated PAL preparation to those sections of the gastrointestinal tract before a direct reaction with phenylalanine conditions, both through the acidic environment of the stomach and through the alkaline environment of the intestine. The dynamics of in vitro degradation of capsules from natural polysaccharides with the PAL enzyme preparation inside in model biological fluids are presented in Figure 1, Figure 2 and Figure 3. In distilled water, the degree of degradation of capsules with PAL after 120 min of the experiment reached 55–65%.

The maximum degradation of capsules with the PAL enzyme preparation (the proportion of capsule weight loss was 90–98%) occurred in model bio-relevant media simulating intestinal juice: SIF and FaSSIF. It should be noted that the capsule degradation rate was higher in the SIF model intestinal juice without pancreatin (the proportion of capsule weight loss was 92–98%, the exposure time was 15 min) compared to the model intestinal juice after fasting FaSSIF (capsule weight loss was 90–97%, exposure time 30 min). In the SGF and FaSSGF media, simulating gastric juice, the proportion of capsule weight loss did not exceed 47% when the exposure time was increased to 90 min and 110 min in SGF and FaSSGF media, respectively.

## 3. Discussion

The transit of dosage forms is quite variable and depends on several factors, such as the time point of dosage, the form of administration, and food intake [48]. The gastrointestinal behavior of acid-resistant hard capsules was described in study [49], and the effect of their density on the time spent in the stomach was determined. The mean gastric emptying time in the systems was 45 ± 35 min for floating DRcaps and 36 ± 18 min for sinking DRcaps. The difference in density did not have a noticeable effect on gastric emptying. In our in vitro study, the destruction of up to 50% of capsules in the acidic model media within the observed period (360 min) was not detected. Depending on the formulation, the destruction of capsules ranged from 26% to 46% at the end of 360 min. Considering that capsules stayed in the stomach for 80 min, 36–45% and 20–30% capsule destruction occurred for the SGF and FaSSGF model media, respectively. Studies have determined that the proportion of capsule weight loss in the acidic environment of the stomach should not exceed 47%. Therefore, we can assume that PAL in the developed capsule form will maintain its stability throughout the entire time spent in the acidic environment of the stomach. However, the results of the examination of patients showed that after oral ingestion in conditions of fasting (at least 8 h without eating), the transit of a single dosage form occurred in a wide time range, lasting from 1 min to 185 min with an average of 21 min. Ingestion of single-unit dosage forms with food or shortly after meals (no more than 30 min) led to a noticeable delay in gastric emptying in the range from 69 min to 583 min with an average value of 302 min. Apparently, the developed encapsulated enzyme preparation PAL should be taken together with food. In this case, capsules made of natural polysaccharides will deliver PAL, preserving its stability, to the upper intestines through the aggressive acidic environment of the stomach. A longer stay of capsules in the stomach does not guarantee that qualitative characteristics of the enzyme will be preserved.

Using magnetic resonance imaging [50], it was found that the advancement rate of the capsule movement decreased with increasing transit time in the small intestine. The highest capsule advancement rate was observed in the measurement interval lasting from 120 to 125 min after taking the capsule. The movement of capsules in the intestine will lead to significant breakdown of the membranes (up to 90%), which will provide the necessary release of the enzyme preparation. In our studies, up to 50% of capsules were destroyed in the intestinal model media within the range of 7 to 15 min. This will allow releasing the enzyme preparation in the upper intestine regions to provide a therapeutic effect. During in vitro experiments to study the degree of degradation of capsules in model media, PAL activity did not undergo significant changes compared to the initial activity. This fact allows us to hope for the possibility of preserving the qualitative characteristics of the enzyme preparation in the gastrointestinal tract. However, additional research is needed to confirm this.

## 4. Materials and Methods

### 4.1. Materials and Chemicals

The object of the study was an encapsulated form based on natural polysaccharides for PAL. Agar-agar (Bio-Rad Laboratories, Hercules, CA, USA), iota-carrageenan (Newgreen Pharmchem Co., Beijing, China), and carboxymethyl cellulose (Acros Organics, Geel, Belgium) were used in this study. Maleic acid (cis-butenedioic acid, toxilic acid, ≥99%, M0375), glycerol (1,2,3-propanetriol, glycerin, ≥99.0%, G5516), potassium phosphate monobasic (monopotassium phosphate, potassium dihydrogen phosphate, prim.-potassium phosphate, ≥99.0%, P5655), sodium hydroxide (caustic soda, ≥98%, S8045), sodium chloride (halite, ≥99.5%, S7653), L-α-phosphatidylcholine (1,2-diacyl-sn-glycero-3-phosphocholine, 3-sn-phosphatidylcholine, L-α-lecithin, azolectin, PC, ≥99%, P3556), sodium taurocholate (taurocholic acid sodium salt, European Pharmacopoeia (EP) Reference Standard, S0900000), pepsin from porcine gastric mucosa (pepsin A, pepsin from hog stomach, 3,200–4,500 U/mg, P6887), hydrochloric acid (1.0 N, BioReagent, suitable for cell culture, H9892) were purchased from Fluka/Sigma-Aldrich (Sigma-Aldrich Rus, Moscow, Russia). All other chemicals (analytical or better grade) used in this study were obtained from the Institute of Biotechnology of Kemerovo State University (Kemerovo, Russia).

### 4.2. Capsule Preparation

Capsule shells for stabilization of L-phenylalanine ammonia-lyase based on aqueous solutions of plant hydrocolloids and glycerol (the use of a plasticizer was necessary to increase flexibility and thereby increase the decay time of the capsule matrix based on natural polysaccharides [51,52]) was studied. The capsule shell formulations (Table 1) were compiled on the basis of our previous studies [46]. The technology for the production of capsules based on plant hydrocolloids included a number of sequential operations: acceptance and quality control of raw materials; preparation of a mixture of plant hydrocolloids, glycerin, and water according to the formulations; mixture stabilization; molding capsule shells; filling the capsule body with contents; capsule drying; capsule quality control; packaging; storage [51,52,53,54,55].

### 4.3. Capsule Quality Analysis

One hundred capsules were examined to determine the weight and diameter of the capsules. Weighing was carried out on a torsion balance (VT-500, MASSA-K, Saint-Petersburg, Russia) with an accuracy of 0.001 g. The diameter of the capsules was measured using the electronic digital micrometer MKTs 25 (LLC “PKP Elbor”, Yekaterinburg, Russia) with an accuracy of 0.001 mm. Each measurement was carried out in triplicate.

The mass fraction of moisture in the capsules was determined according to the standard procedure of OFS 42-0087-08 “Loss in mass upon drying”. For this, the capsules were triturated and placed on filter paper. Samples in filter paper were placed in an oven and dried at 100–110 °C for 1 h, cooled for about 20 min in a desiccator, and weighed. The mass fraction was calculated by the difference in weight translated into percent.

To determine the solubility of the capsules in water, the capsules were kept for 2 h in purified water and their condition was monitored.

The flowability and angle of repose of the capsules were determined on a VP-12a instrument according to standard methods of the State Pharmacopeia XI ed. Flowability is expressed as the average velocity of the outflow of material through the opening of the funnel of a certain diameter. The flowability was determined using a VP-12A model vibration device, the main part of which is a funnel with a 60° cone angle and a nose cut at a right angle at a distance of 3 mm from the end of the funnel cone. The granulate (100.0 g) was placed in a funnel and the exsuction rate was determined. For the reliability of the results obtained, the experiment was carried out 10 times, and calculating the arithmetic mean, we determined the final value, which was 14.1 sec. The flowability of the capsules was determined by the flow rate Vc, calculated using the following formula:

V_c_ = m/t (1)

where, Vc—flowability, g/sec; m—weight of sample, g; t—time of full discharge, sec.

Assessment of microbiological safety indicators for the obtained capsules was carried out in accordance with OFS.1.2.4.0002.15 [48].

### 4.4. Capsule Destruction

The study of the dynamics of in vitro degradation of the encapsulated shell of the enzyme preparation PAL (capsule samples No. 1–6) was carried out in distilled water and model biological fluids using a laboratory identifier of the disintegration process. ERWEKA laboratory identifier ZT 220 series consists of a collection basket, a 1 L liquid vessel, a thermostatic device that maintains the liquid temperature within (37 ± 2) °C, and an electromechanical device that informs the basket of the reciprocating movement in a vertical plane at a frequency of 28–32 cycles in 1 min at a distance of not less than 50 mm and not more than 60 mm. Weighed samples were placed in a solution of 0.1 M hydrochloric acid and kept there for no more than 60 min, then washed with water and immersed in a solution of bicarbonate. After some time, the samples were lowered into a phosphate buffer prepared with citric acid and disodium phosphate, followed by transfer to a solution phosphate buffer, including potassium dihydrogen phosphate and disodium phosphate. When immersed in solutions, capsule behavior was observed. Models of gastric juice SGF without pepsin, model intestinal juice SIF without pancreatin, model fasted intestinal juice (FaSSIF), and model fasted gastric juice (FaSSGF) were used as bio-relevant media imitating intestinal and gastric juices (Table 4).

### 4.5. Statistical Analysis

Each experiment was repeated three times and data were expressed as means ± standard error (SE). Data processing was carried out by standard methods of mathematical statistics. Homogeneity of the sampling effects was checked using the Student’s t-test. The data were subjected to analysis of variance (ANOVA) using Statistica 10.0 (StatSoft Inc., 2007, Tulsa, OK, USA). Differences between means were considered significant when the confidence interval was smaller than 5% (*p* < 0.05).

## 5. Conclusions

The possibility of presenting the enzyme preparation L-phenylalanine ammonia-lyase in capsule form was studied. Natural polysaccharides were used as the base of the capsules. Six formulations of mixtures were developed. The study of the dynamics of degradation under the influence of model media suggested the possibility of using capsules from natural polysaccharides (agar-agar, carrageenan, and carboxymethyl cellulose) as a transport for targeted delivery of the active substance L-phenylalanine ammonia-lyase in the treatment of patients with phenylketonuria. Significant release of the L-phenylalanine ammonium-lyase preparation’s active ingredients from capsules based on plant hydrocolloids is assumed to happen in the upper regions of the small intestine after passing the stomach. However, in order to guarantee the delivery of an effective enzyme preparation, it is necessary to ensure the stability of the PAL inside the capsule shell. The physicochemical properties of natural polysaccharides are different and affect enzyme activity differently. To confirm the proposed assumptions and the possibility of using the developed capsule shells in the manufacture of a phenylketonuria therapy drug, additional studies of the capsulated form of PAL in in vitro and in vivo systems are needed.

## Figures and Tables

**Table 1:** Capsule Shell Formulations.

Formulation No.	The Amount of Component, Mass. %				
	Carrageenan	Agar-agar	CMC*	Glycerol	Water
1	10.0	–	5.0	5.0	80.0
2	5.0	–	10.0	5.0	80.0
3	–	10.0	5.0	5.0	80.0
4	5.0	5.0	–	5.0	85.0
5	10.0	10.0	–	5.0	75.0
6	10.0	10.0	10.0	5.0	65.0

*CMC—carboxymethyl cellulose.

**Table 2:** Physicochemical properties of capsules based on plant hydrocolloids.

Indicator	Capsule Samples					
	1	2	3	4	5	6
Capsule diameter, mm	6.5 ± 0.3^a^	6.2 ± 0.3^a^	6.0 ± 0.3^a^	6.7 ± 0.3^a^	6.9 ± 0.3^a^	6.4 ± 0.3^a^
Capsule length, mm	14.8 ± 0.7^a^	15.0 ± 0.8^a^	14.7 ± 0.7^a^	14.5 ± 0.7^a^	14.4 ± 0.7^a^	14.6 ± 0.7^a^
Useful volume, ml	0.25 ± 0.01^a^	0.24 ± 0.01^a^	0.27 ± 0.01^a^	0.26 ± 0.01^a^	0.23 ± 0.01^a^	0.24 ± 0.01^a^
Capsule weight, mg	42.0 ± 2.1^a^	40.0 ± 2.0^a^	45.0 ± 2.3^a^	43.0 ± 2.2^a^	44.0 ± 2.2^a^	41.0 ± 2.1^a^
Moisture content, %	12.8 ± 0.6^a^	13.5 ± 0.7^a^	12.5 ± 0.6^a^	13.0 ± 0.7^a^	14.2 ± 0.7^b^	13.7 ± 0.7^a^
Disintegration of the capsule, min	16.0 ± 0.8^b^	15.5 ± 0.8^a^	14.0 ± 0.7^a^	15.0 ± 0.7^a^	17.0 ± 0.9^b^	15.0 ± 0.7^a^
Flowability, g/s	8.5 ± 0.4^b^	7.7 ± 0.4^a^	8.0 ± 0.4^a^	8.4 ± 0.4^b^	7.2 ± 0.4^a^	7.0 ± 0.4^a^

* 1—samples of capsules made according to the formulation No. 1; 2—samples of capsules made according to the formulation No. 2; 3—samples of capsules made according to the formulation No. 3; 4—samples of capsules made according to the formulation No. 4; 5—samples of capsules made according to the formulation No. 5; 6—samples of capsules made according to the formulation No. 6. The data are expressed as mean ± standard deviation (*n* = 100). Values followed by same letter (a or b) in a line do not differ significantly (*P* > 0.05) by LSD post hoc test.

**Table 3:** The results of the study of microbiological safety indicators of capsules based on plant hydrocolloids.

Indicator	Capsule Samples					
	1	2	3	4	5	6
QMAFAnM, CFU/g	0.9·10^1^	0.5·10^1^	0.2·10^2^	1.0·10^1^	1.2·10^1^	0.7·10^1^
Yeast and mold, cfu/g	-	-	-	-	-	-
Bacteria of the *E. coli* group, cells in 1 g	-	-	-	-	-	-

* 1—samples of capsules made according to the formulation No. 1; 2—samples of capsules made according to the formulation No. 2; 3—samples of capsules made according to the formulation No. 3; 4—samples of capsules made according to the formulation No. 4; 5—samples of capsules made according to the formulation No. 5; 6—samples of capsules made according to the formulation No. 6.

**Table 4:** Model bio-relevant media.

Medium Designation	Medium Composition
	Intestinal
SIF w/o pancreatin (pH 7.5)	Potassium dihydrogen phosphate—50 mmole Sodium hydroxide—up to pH 7.5
FaSSIF (pH 6.5)	Sodium taurocholate—3 mmole Lecithin—0.2 mmole Maleic acid—19.12 mmole Sodium hydroxide—34.8 mmole Sodium chloride—68.62 mmole
	Gastric
SGF w/o pepsin (pH 1.2)	Sodium chloride—30 mmole Hydrochloric acid—up to pH 1.2
FaSSGF (pH 1.6)	Sodium taurocholate—0.08 mmole Lecithin—0.02 mmole Pepsin with an activity of at least 600 U/mg—0.1 mg/mL Sodium chloride—34.2 mmole Hydrochloric acid—up to pH 1.6
